# Cardiovascular risk of gonadotropin-releasing hormone antagonist versus agonist in men with prostate cancer: an observational study in Taiwan

**DOI:** 10.1038/s41391-022-00555-0

**Published:** 2022-06-03

**Authors:** Yu-Hsuan Joni Shao, Jian-Hua Hong, Chun-Kai Chen, Chao-Yuan Huang

**Affiliations:** 1 Graduate Institute of Biomedical Informatics, College of Medical Science and Technology, Taipei Medical University, Taipei, Taiwan; 2 Clinical Big Data Research Center, Taipei Medical University Hospital, Taipei, Taiwan; 3 Department of Urology, National Taiwan University Hospital, College of Medicine, National Taiwan University, Taipei, Taiwan; 4 Institute of Biomedical Engineering, National Taiwan University, Taipei, Taiwan; 5 Division of Cardiology, Department of Internal Medicine, National Taiwan University Hospital, Hsinchu Branch, Hsinchu, Taiwan

**Keywords:** Cancer therapy, Prostate cancer

## Abstract

**Background:**

The impact of gonadotropin-releasing hormone (GnRH) antagonist and agonist (GnRHa) treatment on cardiovascular disease (CVD) risk in prostate cancer (PCa) remains inconclusive due to conflicting findings. We compared the effects of GnRH antagonist and GnRHa treatments on CVD risk in patients with PCa and pre-existing CVD, in a Taiwan population-based database.

**Methods:**

We assessed the risk of major adverse CV events (MACE: ischemic heart disease [IHD], stroke, congestive heart failure [CHF] or all cause deaths) and composite CV events (IHD, stroke, CHF or CV deaths) occurring ≥90 days after androgen deprivation therapy (ADT) initiation in patients with PCa after 90 days of treatment with either GnRH antagonist (degarelix; *n* = 499) or GnRHa (goserelin, leuprolide, triptorelin; *n* = 15,127). Patients identified with pre-existing CVD had received cardiac therapy for IHD, reported a stroke or CHF within a year before ADT initiation. Adjusted hazard ratios (aHR) and 95% confidence interval (CI) were obtained for MACE and composite CV events risk after adjusting for age, baseline status of diabetes, hypertension and treatments received.

**Results:**

All GnRH antagonist-treated patients showed lower risk of composite CV events than the GnRHa-treated patients. The lower composite CV events risk associated with GnRH antagonist was also observed in patients with metastasis at diagnosis (aHR 0.16; 95% CI, 0.04–0.38; *p* = 0.013) and those receiving ADT for more than six months (aHR 0.30; 95% CI, 0.16–0.54; *p* < 0.0001). In patients with pre-existing CVD, the MACE risk was 33% lower (aHR 0.67; 95% CI, 0.46–0.96; *p* = 0.0299) and composite CV events risk was 84% lower (aHR 0.16; 95% CI, 0.05–0.50; *p* = 0.0017) in GnRH antagonist-treated than the GnRHa-treated patients.

**Conclusions:**

In patients with PCa and pre-existing CVD, GnRH antagonist use was associated with lower risks for composite CV events and MACE compared with GnRHa.

## Introduction

Prostate cancer (PCa) is globally the second most frequently diagnosed cancer in men, ranking after lung cancer [1]. In Taiwan, PCa increased in ranking from eighth to fifth among all cancers in men, with an incidence increase from 1.85% (1988–1992) to 8.96% (2013–2016) [2]. The PCa mortality rates in Taiwanese men increased from 8.3 to 11.5 per 100,000 people between 2006 and 2016 [3]. Among the non-cancer mortality causes in patients with PCa, cardiovascular disease (CVD) is the leading cause of death in the United States and many other countries [4–6]. The proportion of CVD fatalities increases over time after diagnosis, especially in those on long-term androgen deprivation therapy (ADT) [7].

ADT is the primary systemic therapy (standalone/concomitant) in PCa treatment for advanced disease. It reduces testosterone levels to those achieved by castration [8]. Approximately 50% of patients with PCa receive ADT during their treatment [9]. Androgen deprivation is achieved by gonadotropin-releasing hormone (GnRH) antagonists or agonists (GnRHa), which prevent luteinizing hormone (LH) secretion, and consequently inhibit testosterone production [10].

Reports of the relative associations between CVD and, GnRH antagonist/GnRHa are conflicting, especially in PCa patients with pre-existing CVD risk [11, 12]. Emerging literature suggests lower major adverse cardiovascular events (MACE) in patients treated with GnRH antagonists but remains inconclusive in patients with pre-existing CVD risk [12–18]. GnRHa has been associated with an increased CVD risk and related mortality, including in patients with PCa and prior CVD [13, 14, 16].

Clinical trials assessing the differential effect of GnRH antagonists and GnRHa on CVD remain inconclusive [15, 18–20]. Observational studies report a lower risk of CVD with GnRH antagonists than GnRHa [12, 14, 17]. However, the differential effect of GnRH antagonists or GnRHa monotherapy, without switching between drugs, is scarcely reported in Asian patients. Therefore, through this real-world data (RWD) analysis, we aim to compare the impacts of a GnRH antagonist (degarelix) and GnRHa treatments (goserelin, leuprolide, triptorelin), on the CVD risk in patients with PCa and pre-existing CVD, using Taiwan National Health Insurance Research Data (NHIRD).

## Methods

### Data source and study population

This population-based cohort study used NHIRD, which is linked by encrypted patient identifiers to the Taiwan Cancer Registry (TCR). The NHIRD includes medical claims data of demographics, enrollment profiles, disease diagnosis, procedures, and drug prescriptions; the TCR covers 97% of cancer cases in Taiwan and has a good level of data accuracy [13, 21, 22]. NHIRD are additionally linked to the Death Registry, to ascertain vital status and cause of death [23].

Patients, aged ≥20 years, with PCa (*n* = 18,835), who, according to prescription records, initiated ADT between January 1, 2015 and December 31, 2019, and sustained this for ≥3 months, were identified in the TCR, using the International Classification of Disease (ICD-O-3) code, C61.9. Patients who had a recorded orchiectomy (*n* = 146) or had received both GnRH antagonist and GnRHa for ≥3 months (*n* = 3063) were excluded.

Of eligible patients, GnRH antagonist exposure included patients treated with degarelix (*n* = 499; Anatomical Therapeutic Chemical Classification System [ATC] code: L02BX02), patients receiving GnRHa included goserelin, leuprolide and triptorelin treatment (*n* = 15,127; ATC codes: L02AE03, L02AE02, and L02AE04 respectively). Concomitant treatment data were extracted for: antiandrogens (bicalutamide, cyproterone, and flutamide; ATC codes: L02BB03, G03HA01 and L02BB01 respectively), estrogens (ATC code: L02AA), ketoconazole (ATC code: J02AB02), and androgen receptor (AR)-directed therapy for new-generation drugs (abiraterone, enzalutamide; ATC codes: L02BX03, L02BB04 respectively). Data were also extracted for chemotherapy and radiation therapy. The institutional review board of Taipei Medical University reviewed and approved this study (TMU-JIRB no. 201502042).

### Covariates

For pre-existing CVD assessment, the following covariates were considered: hypertension (≥2 diagnoses within six months), cardiac therapy (≥2 prescriptions for drugs with ATC code: C01), acute myocardial infarction and other forms of ischemic heart disease (IHD), stroke, congestive heart failure (CHF), diabetes (≥2 diagnoses within six months), dyslipidemia (≥2 diagnoses within six months) and death (Supplementary Table 1).

The pre-existing CVD was assessed at baseline; patients receiving cardiac therapy, having a diagnosis of IHD, stroke or CHF 1 year before ADT initiation were categorized as having pre-existing CVD. Demographic and clinical characteristics were recorded at baseline. Additional covariates included age at diagnosis, clinical disease stage, Gleason score, prostate-specific antigen (PSA) level at diagnosis, and comorbidities. Patients’ ages were grouped as ≤54, 55–59, 60–69, 70–74, and ≥75 years.

The clinical stage was classified based on the TNM stage using the American Joint Committee on Cancer classification system (T scoring for area and size to give the extent of the main tumor: 1, 2, 3, 4 and Missing; N, for spread to lymph nodes, and M, for metastasis, both scoring: 0 or 1) [24]. Cancers were graded on differentiation using Gleason score (GS) and categorized based on score ranges 2–6, 7, and 8–10 [25]. The PSA (ng/mL) concentrations at diagnosis were also categorized as ≤50 ng/mL, >50 ng/mL and “missing”. To assess the burden of comorbidities and association with survival, the Charlson’s comorbidity index (CCI) was recorded at baseline, including assessment of claims during 1 year before the PCa diagnosis, excluding cancer diagnoses [26].

Patients were stratified based on assessed cancer risk, using the National Comprehensive Cancer Network (NCCN) staging, which considers: the clinical T stage, pre-treatment PSA level, and the GS (Table 1 footnote) [27]. The resulting risk groups allowed for comparison across patients with the same prognosis.

**Table 1:** Baseline demographics and clinical characteristics.

	GnRH antagonist *N* = 499	GnRH agonist *N* = 15,127	
	*n* (%)	*n* (%)	*P* value
Age (years)			0.4557
≤54	11 (2.2)	280 (1.9)	
55–59	18 (3.6)	655 (4.3)	
60–69	127 (25.5)	4037 (26.7)	
70–74	87 (17.4)	2953 (19.5)	
≥75	256 (51.3)	7202 (47.6)	
Cancer TNM staging			<0.0001
Localized	132 (26.5)	5905 (39.0)	
Locally advanced	74 (14.8)	2743 (18.1)	
Any T, N ≥ 1	39 (7.8)	1218 (8.05)	
Any T, any N and M ≥ 1	254 (50.9)	5261 (34.8)	
Gleason score			0.0002
2–6	41 (8.2)	2032 (13.4)	
7	129 (25.9)	4512 (29.8)	
8–10	313 (62.7)	8086 (53.5)	
Missing	16 (3.2)	497 (3.3)	
PSA, ng/mL			0.0256
≤50	10 (2.0)	501 (3.3)	
>50	450 (90.2)	13,006 (86.0)	
Missing	39 (7.8)	1620 (10.7)	
Risk groups			<0.0001
Low–intermediate	38 (7.6)	1698 (11.2)	
High–very high	121 (24.3)	5314 (35.1)	
Regional or metastatic	340 (68.1)	8115 (53.7)	
CCI			< 0.0001
0	90 (18.0)	4266 (28.2)	
1	142 (28.5)	4273 (28.3)	
2	117 (23.5)	2590 (17.1)	
3+	150 (30.1)	3998 (26.4)	
Concomitant medication			
Antiandrogen	90 (18.0)	7489 (49.5)	<0.0001
Abiraterone	21 (4.2)	1058 (7.0)	0.0009
Bicalutamide	165 (33.1)	10,023 (66.3)	<0.0001
Cyproterone	56 (11.2)	4489 (29.7)	<0.0001
Enzalutamide	17 (3.4)	746 (4.9)	0.0542
Flutamide	12 (2.4)	1490 (9.9)	<0.0001
Pre-existing cardiovascular disease factors			
Ischemic heart disease	35 (7.1)	882 (5.8)	0.2685
Congestive heart failure	10 (2.0)	155 (1.0)	0.0352
Stroke	11 (2.2)	245 (1.6)	0.3113
Hyperlipidemia	87 (17.4)	1694 (11.2)	<0.0001
Hypertension	128 (25.7)	3557 (23.5)	0.2685
Diabetes	58 (11.6)	1486 (9.8)	0.1850
Use of cardiac therapy	92 (18.4)	1580 (10.4)	<0.0001
Pre-existing CVD			<0.0001
Yes^a^	167 (33.5)	3348 (22.1)	
No	332 (66.5)	11,779 (77.9)	

Risk groups defined by National Comprehensive Cancer Network guidelines were as follows:

Low risk: T1–T2a, GS ≤ 6, and PSA of 10  ng/mL; favorable intermediate risk: T2b–T2c or GS ≤ 7 or PSA of 10–20  ng/mL and a primary GS of 3; unfavorable intermediate risk: T2c or GS ≤ 7 or PSA of 10–20 ng/mL and primary GS of 4; high risk: T3a or GS of 8–10 or PSA >20 ng/mL; very high risk for locally advanced prostate cancer: T3b–T4 or primary GS component scores of ≥5 scores with overall GS of 8–10; metastatic risk: N1 or M1 with any T stage [29].

*CCI* Charlson comorbidity index, *CVD* cardiovascular disease(s), *GnRH* gonadotropin-releasing hormone, *PSA* prostate specific antigen, *TNM* where T = tumor, N = nodes and M = metastasis.

^a^Yes: receiving cardiac therapy, diagnosis of ischemic heart diseases, stroke, or congestive heart failure 1 year before androgen deprivation therapy initiation.

### Outcome variables

Primary outcomes were MACE defined as IHD, stroke, CHF or all cause deaths occurring ≥90 days after ADT initiation, whichever came first. Secondary outcomes were composite CV events including IHD, stroke, CHF or CV deaths occurring ≥90 days after ADT initiation, whichever came first. Death Registry-recorded causes of death were used to determine CV deaths, defined by ICD-10 using I00–I99.

### Statistical analysis

The baseline characteristics, MACE and composite CV events risk of the GnRH antagonist and GnRHa groups, also the pre-existing CVD and no pre-existing CVD subgroups were tested for significant difference by Chi-square test or Fisher’s exact test for categorical variables. The Cox proportional hazard model was used to estimate the differential of MACE associated with GnRH antagonist compared with GnRHa. The Fine and Gray hazard model, which considers non-CV deaths as competing risks in deriving the event probability over time, was employed to assess the risk of composite CV events [28, 29]. The hazard ratio (HR) with 95% confidence interval (CI) was calculated and adjusted for age, cancer stage, or receiving radiation therapy, chemotherapy, antiandrogen, abiraterone, and enzalutamide. The Kaplan–Meier survival curves were plotted for all patients as well as in subgroups of pre-existing CVD for MACE-free survival and composite CV event-free survival, for GnRH antagonist and GnRHa-treated patients. Log-rank tests were performed to test the differences in survival between treatment groups. Statistical analysis was performed using SAS version 9.4 (SAS Institute, Cary, NC, USA). Statistical significance is indicated by a *p* value of <0.05.

## Results

### Baseline characteristics

A total of 499 patients were included in the GnRH antagonist group and 15,127 patients in the GnRHa group. The mean (standard error) survival time for all patients was 2.62 (1.49) years. Patients from both groups were of similar ages (*p* = 0.4557), with the largest proportion of both groups being aged ≥75 years (Table 1).

A significantly higher proportion of patients had clinically advanced cancer stage in the GnRH antagonist treatment group (based on cancer stage, higher cancer grade, increased PSA and had “regional or metastatic” disease spread; Table 1). The proportion of patients with comorbidities was higher in the GnRH antagonist than the GnRHa group (Table 1). At baseline, a greater proportion of patients treated with GnRH antagonist had pre-existing CVD compared with the GnRHa-treated patients (33.5% vs 22.1% respectively, *p* < 0.0001; Table 1). A significantly lower proportion of patients from the GnRH antagonist than GnRHa group received baseline concomitant medications: antiandrogens (*p* < 0.0001), abiraterone (*p* = 0.0009), bicalutamide (*p* < 0.0001), cyproterone (*p* < 0.0001), and flutamide (*p* < 0.0001; Table 1).

### Subgroup comparison

#### Cardiovascular outcomes

After 90 days of ADT, the proportions of patients receiving hyperlipidemia or cardiac treatments were significantly lower in the GnRH antagonist than GnRHa-treated group (hyperlipidemia treatment: 25.1% vs 34.3%, respectively; *p* < 0.0001; cardiac therapy: 24.6% vs 32.8% respectively; *p* < 0.0001). A similar trend was observed across both groups with pre-existing CVD (hyperlipidemia treatment: 36.5% vs 47.5%, respectively; *p* = 0.0057; cardiac therapy: 33.5% vs 41.3% respectively; *p* = 0.0478) and no pre-existing CVD (hyperlipidemia treatment: 19.3% vs 30.5% respectively; *p* < 0.0001; cardiac therapy: 20.2% vs 30.4% respectively; *p* < 0.0001; Table 2). Similarly, IHD, stroke, or CHF were observed in a significantly lower proportion of patients from the GnRH antagonist-treated group than the GnRHa-treated group (3.6% vs 11.6% respectively; *p* < 0.0001) and across both groups with pre-existing CVD (1.8% vs 12.3% respectively; *p* < 0.0001) and no pre-existing CVD (4.5% vs 11.4% respectively; *p* < 0.0001; Table 2). However, no significant difference in CV-related or other-cause deaths was observed among the groups.

**Table 2:** Cardiovascular outcomes (after 90 days of ADT initiation) among patients on GnRH antagonists and agonists by pre-existing cardiovascular disease.

	All			Pre-existing CVD^a^			No pre-existing CVD		
	GnRH antagonist *N* = 499	GnRH agonist *N* = 15,127		GnRH antagonist *N* = 167	GnRH agonist *N* = 3348		GnRH antagonist *N* = 332	GnRH agonist *N* = 11,779	
	*n* (%)	*n* (%)	*P* value	*n* (%)	*n* (%)	*P* value	*n* (%)	*n* (%)	*P* value
Receiving hyperlipidemia treatment	125 (25.1)	5183 (34.3)	<0.0001	61 (36.5)	1589 (47.5)	0.0057	64 (19.3)	3594 (30.5)	<0.0001
Receiving cardiac therapy	123 (24.6)	4960 (32.8)	<0.0001	56 (33.5)	1381 (41.3)	0.0478	67 (20.2)	3579 (30.4)	<0.0001
IHD, stroke or CHF	18 (3.6)	1758 (11.6)	<0.0001	3 (1.8)	410 (12.3)	<0.0001	15 (4.5)	1348 (11.4)	<0.0001
Vital status			0.3939			0.6542			0.6231
Alive	377 (75.6)	11,229 (74.2)		130 (77.8)	2570 (76.8)		247 (74.3)	8659 (73.5)	
CV-related death	7 (1.4)	348 (2.3)		2 (1.2)	76 (2.3)		5 (1.5)	272 (2.3)	
Other cause death	115 (23.0)	3550 (23.5)		35 (21.0)	702 (21.0)		80 (24.1)	2848 (24.2)	

*ADT* androgen deprivation therapy*, CHF* congestive heart failure, *CV* cardiovascular, *GnRH* gonadotropin-releasing hormone, *IHD* ischemic heart disease.

^a^Pre-existing CVD: receiving cardiac therapy, diagnosis of ischemic heart diseases, stroke, or congestive heart failure 1 year before androgen deprivation therapy initiation.

#### MACE and composite CV events risk outcomes

MACE risk was not significantly different between the GnRH antagonist and GnRHa-treated patients. However, a significantly lower MACE risk was determined for patients with pre-existing CVD in the GnRH antagonist group than the GnRHa-treated group (adjusted hazard ratio [aHR] 0.67; 95% CI, 0.46–0.96; *p* = 0.0299; Table 3). In the no pre-existing CVD group, the MACE risk was not significantly different between the GnRH antagonist and GnRHa-treated groups (Table 3). The composite CV events risk was significantly lower in GnRH antagonist‍-‍treated patients than the GnRHa-treated patients (aHR 0.34; 95% CI, 0.21‍–‍0.55; *p* < 0.0001, Table 3). Similar lower risk of composite CV events was determined in GnRH antagonist-treated patients across the pre-existing (aHR 0.16; 95% CI, 0.05‍–‍0.50; *p* = 0.0017, Table 3) and no-pre-existing CVD (aHR 0.44; 95% CI, 0.26‍–‍0.74; *p* = 0.0019, Table 3) group than GnRHa-treated patients.

**Table 3:** Risk of MACE and composite CV events in patients on GnRH antagonists compared with patients on GnRH agonists by pre-existing cardiovascular disease.

	MACE				Composite CV events^a^			
	No. of events	aHR^b^	(95% CI)	*P* value	No. of events	aHR^c^	(95% CI)	*P* value
All								
GnRH antagonist (*n* = 499)	136	0.97	(0.81–1.17)	0.7665	24	0.34	(0.21–0.55)	<0.0001
GnRH agonist (*n* = 15,127)	5105	–			2001	–		
Pre-existing CVD^d^								
GnRH antagonist (*n* = 167)	39	0.67	(0.46–0.96)	0.0299	5	0.16	(0.05–0.50)	0.0017
GnRH agonist (*n* = 3348)	1046	–			462	–		
No pre-existing CVD								
GnRH antagonist (*n* = 332)	97	1.23	(0.91–1.40)	0.2901	19	0.44	(0.26–0.74)	0.0019
GnRH agonist (*n* = 11,779)	4059	–			1539	–		

*aHR* adjusted hazard ratio, *CV* cardiovascular, *CVD*, cardiovascular disease(s), *GnRH* gonadotropin-releasing hormone, *MACE* major adverse cardiovascular event (ischemic heart disease, stroke, congestive heart failure or all cause deaths), occurring ≥90 days after ADT initiation, whichever came first.

^a^Composite CV events: ischemic heart disease, stroke, congestive heart failure or CV deaths occurring ≥90 days after ADT initiation, whichever came first.

^b^Adjusted hazard ratios were estimated using cox model adjusted for age, cancer stage, receiving chemotherapy, radiation therapy, antiandrogen, abiraterone, and enzalutamide.

^c^Adjusted hazard ratios were estimated using the Fine and Gray competing risk model adjusted for age, cancer stage, receiving chemotherapy, radiation therapy, antiandrogen, abiraterone, and enzalutamide.

^d^Pre-existing CVD: receiving cardiac therapy, diagnosis of ischemic heart diseases, stroke, or congestive heart failure 1 year before androgen deprivation therapy initiation.

In patients with a pre-existing CVD and metastasis at diagnosis, the MACE risk was observed to be similar between patients treated with GnRH antagonist and GnRHa (aHR 0.98; 95% CI, 0.66–1.45; *p* = 0.9071; Table 4). Similarly, in patients with pre-existing CVD and receiving ADT for ≥6 months, no significant difference in MACE risk was observed between patients treated with GnRH antagonist and GnRHa (aHR 0.95; 95% CI, 0.74–1.22; *p* = 0.7023; Table 4). The risk of composite CV events was 84% lower in patients with pre-existing CVD and metastasis at diagnosis treated with GnRH antagonists than GnRHa (aHR 0.16; 95% CI, 0.04–0.38; *p* = 0.0130; Table 4). In patients with pre-existing CVD and receiving ADT for ≥6 months, a 88% lower risk of composite CV events was determined in GnRH antagonist treated patients than GnRHa-treated patients (aHR 0.12; 95% CI, 0.03–0.49; *p* < 0.0001; Table 4).

**Table 4:** Subgroup analysis estimating the risk of MACE associated with GnRH antagonist comparing with GnRH agonist.

GnRH antagonist vs GnRH agonist	MACE				Composite CV events			
	No of event	aHR^a^	(95% CI)	*P* value	No of event	aHR	(95% CI)	*P* value
Pre-existing CVD, initial staging *N* = 1 or *M* = 1								
GnRH antagonist (*n* = 106)	34	0.98^a^	(0.66–1.45)	0.9071	3	0.16^b^	(0.04–0.38)	0.013
GnRH agonist (*n* = 1489)	621				188			
Receiving more than 6 months of ADT (GnRH antagonist ≥6 months vs GnRH agonist ≥6 months)								
GnRH antagonist (*n* = 286)	82	0.95	(0.74–1.22)	0.7023	15	0.30	(0.16–0.54)	<0.0001
GnRH agonist (*n* = 10,615)	3780				1637			
Pre-existing CVD, receiving more than 6 months of ADT (GnRH antagonist ≥6 months vs GnRH agonist ≥6 months)								
GnRH antagonist (*n* = 96)	24	0.64^c^	(0.39–1.05)	0.0757	3	0.12^d^	(0.03–0.49)	0.0032
GnRH agonist (*n* = 2006)	687				375			

*aHR* adjusted hazard ratio, *CV* cardiovascular, *GnRH* gonadotropin-releasing hormone*, MACE* major adverse cardiovascular event (ischemic heart disease, stroke, congestive heart failure or CV-related death). preexising CV risk: receiving cardiac therapy, diagnosis of ischemic heart diseases, stroke, or congestive heart failure 1 year before androgen deprivation therapy initiation.

^a^aHRs were estimated using cox model adjusted for age, receiving chemotherapy, radiation therapy, antiandrogen, abiraterone, and enzalutamide.

^b^aHRs were estimated using the Fine and Gray competing risk model adjusted for age receiving chemotherapy, radiation therapy, antiandrogen, abiraterone, and enzalutamide.

^c^aHRs were estimated using cox model adjusted for age, cancer stage, receiving chemotherapy, radiation therapy, antiandrogen, abiraterone, and enzalutamide.

^d^aHRs were estimated using the Fine and Gray competing risk model adjusted for age, cancer stage, receiving chemotherapy, radiation therapy, antiandrogen, abiraterone, and enzalutamide.

### Survival analysis

**Fig. 1:**
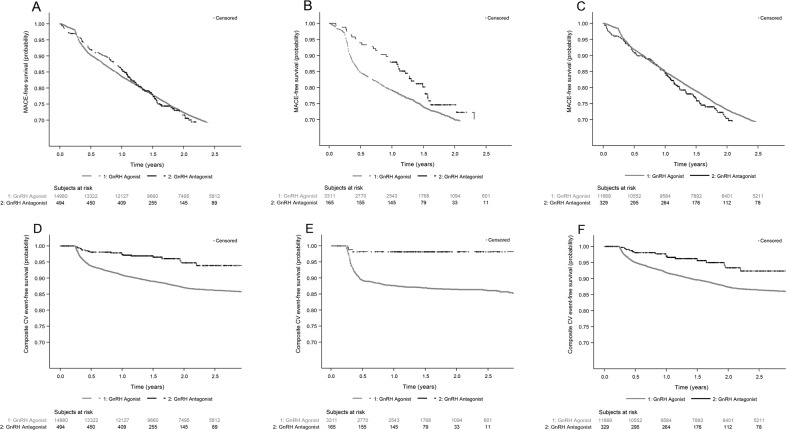
MACE-free survival and composite CV event-free survival after GnRH antagonist or GnRH agonist treatment. Survival curves showing MACE-free survival probability (significance of difference tested by log rank test) in (**A**) all patients (*p* = 0.9569), (**B**)‍ patients with pre-existing CVD (*p* = 0.1029), and (**C**) patients with no pre-existing CVD (*p*‍ = 0.4228); composite CV event-free survival probability in (**D**) all patients (*p* < 0.0001), (**E**)‍ patients with pre-existing CVD (*p* < 0.0001), and (**F**) patients with no pre-existing CVD (*p*‍ = 0.0010). CV cardiovascular, CVD cardiovascular disease(s), GnRH gonadotropin-releasing hormone, MACE major adverse cardiovascular event (ischemic heart disease, stroke, congestive heart failure or all cause deaths), occurring ≥90 days after ADT initiation, whichever came first. Composite CV events: ischemic heart disease, stroke, congestive heart failure or CV deaths occurring ≥90 days after ADT initiation, whichever came first.

MACE-free survival probability was similar across GnRH antagonist and GnRHa-treated patients (*p* = 0.9569) as well as in patients across the pre-existing CVD (*p* = 0.1029), and no pre-existing CVD (*p* = 0.4228) group of patients (Fig. 1A–C). The composite CV event-free probability was significantly higher in GnRH antagonist-treated than GnRHa-treated patients (*p* < 0.0001, Fig. 1D). Similarly, the composite CV event-free survival probability was significantly higher in patients treated with GnRH antagonists with pre-existing CVD (*p* < 0.0001) as well as no pre-existing CVD (*p* = 0.0010) than GnRHa-treated patients (Fig. 1E, F).

## Discussion

This population-based study demonstrated that patients with PCa and pre-existing CVD treated with GnRH antagonist had a lower MACE risk (including IHD, stroke, CHF, or death), than those treated with GnRHa. A lower risk of composite CV events was observed in all GnRH antagonist-treated patients than GnRHa-treated patients, with a lowest risk determined in patients with pre-existing CVD. This is one of the few RWD studies, focusing on patients with pre-existing CVD, which demonstrates better CV outcomes in PCa treated with GnRH antagonist than GnRHa [13, 30–32]. This RWD provides important evidence for consideration in clinical practice, where patients receive ADT for long durations.

PRONOUNCE was the first global randomized trial with blinded adjudication of CV outcomes, in patients with PCa and recorded no difference in MACE risk in patients with known CVD, between degarelix (GnRH antagonist) and leuprolide (GnRHa). However, these results are inconclusive due to study limitations. Several studies have reported an association between GnRH antagonist and a lower risk of CV events compared with GnRHa treatment [13, 14, 16, 17, 33–35], but inclusion of baseline pre-existing CVD status was infrequently reported [13, 30–32].

We report lower MACE risk in patients treated with GnRH antagonist than GnRHa (aHR 0.67; 95% CI, 0.46–0.96; *p* = 0.0299) with pre-existing CVD. Our results are similar to those reported by Albertsen et al. and Margel et al. [14, 16]. We report 84% lower risk of composite CV events in patients treated with GnRH antagonist than GnRHa (aHR 0.16; 95% CI, 0.05–0.50; *p* = 0.0017) with pre-existing CVD. In a study using the same database that we used, but with a different study design, Chen et al. report a 52% lower risk of composite CV events (HR 0.48; 95% CI, 0.25–0.90; *p* = 0.0222) in patients with PCa treated with GnRH antagonist than GnRHa, at 12 months; the risk of CV events was not significantly lower in patients who had myocardial infarction, ischemic stroke, or CVD [13]. Differences in study design may explain the different findings (our study included patients who received ≥3 months GnRH antagonist or GnRHa monotherapy and had a longer treatment duration). Most, but not all RWD suggest fewer CVD events associated with GnRH antagonists than GnRHa [11, 13, 33, 34, 36, 37]. Conflicting results have been attributed to variation in baseline CVD, treatment duration, number of patients, methodological differences and CV outcomes not being the primary study outcome [17]. A Scottish Cancer Registry data analysis showed an increased CVD in patients treated with both GnRH antagonist (HR 1.5; 95% CI, 1.2–1.9) and GnRHa (HR 1.3; 95% CI, 1.2–1.4), compared with untreated patients with PCa; fewer patients were treated with GnRH antagonist than GnRHa, and there was limited adjustment for cancer stage [36]. In a multi-country (UK, Scotland, Belgium, Netherlands and France) RWD analysis and a French RWD analysis, no differences in CVD profiles were observed between the two treatment groups [11, 37]. Switching therapies during the study period, data limitations and small patient numbers on GnRH antagonist may explain these observations. Additionally, baseline CVD status was not considered in these previous studies.

Many conditions and risk factors are identified as contributing to CVD risks in patients with PCa. Therefore, several guidelines and statements have suggested a baseline CVD assessment before starting potentially cardiotoxic cancer treatments [9, 12, 38–40]. In our study, the definition of baseline CVD risk was based on review of codes used in previously published literature for claims-based studies [41]. However, cardiac biomarkers and lifestyle CVD risk factors measured before treatments may effectively manage patients during ADT. Patients with PCa reportedly present a burden of underassessed and undertreated CVD risk factors, including those receiving ADT [42]. Several CVD assessment methods are currently used for risk stratification, but that is not standardized [39, 43]. Intensive research to validate and refine risk stratification methods would mitigate treatment-related CVD risk and improve overall survival [44].

Both GnRH antagonist and GnRHa lead to castration-equivalent testosterone levels by different pathways [8, 33]. Antagonists bind directly to the GnRH receptors and in turn suppress LH and follicle-stimulating hormone (FSH) and reduce testosterone without causing any surge. GnRHa stimulate LH and FSH possibly causing a testosterone microsurge and subsequent receptor desensitization, resulting in castrate testosterone levels [14, 33]. The microsurges caused by GnRHa may have harmful CV effects, and the absence of these is advantageous with GnRH antagonist treatment [19, 45, 46].

Challa et al. hypothesize the possible reasons for GnRHa-related increased CV risks as: low FSH suppression (facilitates atheroma formation/progression), monocyte and T lymphocyte activation, and potentially testosterone microsurges [45]. Together, these actions may promote atherosclerotic plaque formation, disruption, and thrombosis [45]. In patients with pre-existing CVD, the differential effects of GnRH antagonist and GnRHa on MACE and composite CV events may be attributed to GnRHa destabilizing the preestablished atherosclerotic plaques [8, 14]. Proinflammatory T-helper 1 cells are dominant in atherosclerotic plaques; their lymphocytes express GnRH receptors and on activation by the GnRHa lead to T-cell proliferation [8, 14, 33, 44, 47, 48]. T lymphocytes may cause atherosclerotic plaque rupturing through release of proinflammatory cytokines and macrophage stimulation [44]. Macrophages maintain the local inflammatory response, increasing plaque development and thrombosis [49].

An increased invasive ability of macrophages has been reported in the presence of GnRHa, but not GnRH antagonist [13]. Lifshitz et al. hypothesize that in patients with pre-existing CVD, GnRH antagonist treatment has a direct protective effect on plaque stability in contrast to the plaque instability caused by GnRHa treatment. In patients with pre-existing CVD, GnRH antagonist treatment increased serum levels of five proteins associated with plaque stabilizing: human chitotriosidase, macrophage receptor with collagenous structure, cathepsin D, superoxide dismutase-2 and hydroxyacid oxidase-1 [50]. The microenvironment in patients with PCa with pre-existing CVD is altered with distinct lymphocyte, monocyte, and inflammatory modulation, enabling GnRH antagonist treatment to potentially have beneficial effects in patients with pre-existing CVD over GnRHa [50].

The prevalence of CVD is lower in Taiwan than in Western populations [51]. Therefore, treatment-related cardiotoxicity may be underestimated in patients with PCa. The decreased risk of composite CV events and MACE in patients with pre-existing CVD, treated with degarelix in this study population, provides important real-world evidence for physicians, highlighting relevant considerations regarding cardiotoxicity to cancer treatment.

Our study has a few limitations, including lack of body mass index and physical activity data-relevant for CVD baseline context. The number of patients treated with GnRH antagonist was lower than that of GnRHa. We have analyzed the RWD with distinct pre-existing CVD stratification and report the safety of degarelix (GnRH antagonist), including in patients with pre-existing CVD, adding to the limited data in Asian patients with PCa. Our study allows a true comparison between GnRH antagonist and GnRHa treatment, as patients received these single treatments, without overlap.

ADT is the main treatment in advanced stage PCa, and patients are potentially treated over a long duration, so our results provide additional valuable insight evaluating GnRH antagonist and GnRHa as treatment options. Given that PCa incidence rates increase with age, and CVD risk also becomes more likely, GnRH antagonist may provide a safer CV-risk profile for long-term PCa treatment.

## Conclusion

GnRH antagonist reduced risk of MACE and composite CV events in patients with PCa and with pre-existing CVD, relative to GnRHa-treated patients. This effect was most pronounced in GnRH antagonist-treated patients with pre-existing CVD, metastasis at diagnosis and use of ADT ≥ 6 months. These real-world findings are relevant when considering the long-term treatment of PCa.

### Supplementary information

Supplementary table 1

## Data Availability

This study was conducted using the Taiwan National Health Insurance Research Data (NHIRD) database. Due to legal restrictions imposed by the government of Taiwan in relation to the “Personal Information Protection Act”, data cannot be made publicly available. Requests for data can be sent as a formal proposal to the Health and Welfare Data Science Center, Taiwan.
