# Correction to: Cardiovascular risk of gonadotropin-releasing hormone antagonist versus agonist in men with prostate cancer: an observational study in Taiwan

**DOI:** 10.1038/s41391-022-00568-9

**Published:** 2022-07-11

**Authors:** Yu-Hsuan Joni Shao, Jian-Hua Hong, Chun-Kai Chen, Chao-Yuan Huang

**Affiliations:** 1 Graduate Institute of Biomedical Informatics, College of Medical Science and Technology, Taipei Medical University, Taipei, Taiwan, ROC; 2 Clinical Big Data Research Center, Taipei Medical University Hospital, Taipei, Taiwan, ROC; 3 Department of Urology, National Taiwan University Hospital, College of Medicine, National Taiwan University, Taipei, Taiwan, ROC; 4 Institute of Biomedical Engineering, National Taiwan University, Taipei, Taiwan, ROC; 5 Division of Cardiology, Department of Internal Medicine, National Taiwan University Hospital, Hsinchu Branch, Hsinchu, Taiwan, ROC

**Keywords:** Cancer therapy, Prostate cancer

Correction to: *Prostate Cancer and Prostatic Diseases* 10.1038/s41391-022-00555-0, published online 3 June 2022

In this article, author names Yu-Hsuan Joni Shao, Jian-Hua Hong, Chun-Kai Chen, and Chao-Yuan Huang were incorrectly written as Shao Yu-Hsuan Joni, Hong Jian-Hua, Chen Chun-Kai, and Huang Chao-Yuan.

Tables 1 and 2 have been adjusted to reflect consistency within the paper. They now show as seen below.

**Table 1:** Baseline demographics and clinical characteristics.

	GnRH antagonist *N* = 499	GnRH agonist *N* = 15,127	
	*n* (%)	*n* (%)	*P* value
Age (years)			0.4557
≤54	11 (2.2)	280 (1.9)	
55–59	18 (3.6)	655 (4.3)	
60–69	127 (25.5)	4037 (26.7)	
70–74	87 (17.4)	2953 (19.5)	
≥75	256 (51.3)	7202 (47.6)	
Cancer TNM staging			<0.0001
Localized	132 (26.5)	5905 (39.0)	
Locally advanced	74 (14.8)	2743 (18.1)	
Any T, N ≥ 1	39 (7.8)	1218 (8.05)	
Any T, any N and M ≥ 1	254 (50.9)	5261 (34.8)	
Gleason score			0.0002
2–6	41 (8.2)	2032 (13.4)	
7	129 (25.9)	4512 (29.8)	
8–10	313 (62.7)	8086 (53.5)	
Missing	16 (3.2)	497 (3.3)	
PSA, ng/mL			0.0256
≤50	10 (2.0)	501 (3.3)	
>50	450 (90.2)	13,006 (86.0)	
Missing	39 (7.8)	1620 (10.7)	
Risk groups			<0.0001
Low–intermediate	38 (7.6)	1698 (11.2)	
High–very high	121 (24.3)	5314 (35.1)	
Regional or metastatic	340 (68.1)	8115 (53.7)	
CCI			<0.0001
0	90 (18.0)	4266 (28.2)	
1	142 (28.5)	4273 (28.3)	
2	117 (23.5)	2590 (17.1)	
3+	150 (30.1)	3998 (26.4)	
Concomitant medication			
Antiandrogen	90 (18.0)	7489 (49.5)	<0.0001
Abiraterone	21 (4.2)	1058 (7.0)	0.0009
Bicalutamide	165 (33.1)	10,023 (66.3)	<0.0001
Cyproterone	56 (11.2)	4489 (29.7)	<0.0001
Enzalutamide	17 (3.4)	746 (4.9)	0.0542
Flutamide	12 (2.4)	1490 (9.9)	<0.0001
Pre-existing cardiovascular disease factors			
Ischemic heart disease	35 (7.1)	882 (5.8)	0.2685
Congestive heart failure	10 (2.0)	155 (1.0)	0.0352
Stroke	11 (2.2)	245 (1.6)	0.3113
Hyperlipidemia	87 (17.4)	1694 (11.2)	<0.0001
Hypertension	128 (25.7)	3557 (23.5)	0.2685
Diabetes	58 (11.6)	1486 (9.8)	0.1850
Use of cardiac therapy	92 (18.4)	1580 (10.4)	<0.0001
Pre-existing CVD			<0.0001
Yes^a^	167 (33.5)	3348 (22.1)	
No	332 (66.5)	11,779 (77.9)	

Risk groups defined by National Comprehensive Cancer Network guidelines were as follows:

Low risk: T1–T2a, GS ≤ 6, and PSA of 10  ng/mL; favorable intermediate risk: T2b–T2c or GS ≤ 7 or PSA of 10–20  ng/mL and a primary GS of 3; unfavorable intermediate risk: T2c or GS ≤ 7 or PSA of 10–20 ng/mL and primary GS of 4; high risk: T3a or GS of 8–10 or PSA >20 ng/mL; very high risk for locally advanced prostate cancer: T3b–T4 or primary GS component scores of ≥5 scores with overall GS of 8–10; metastatic risk: N1 or M1 with any T stage [29].

*CCI* Charlson comorbidity index, *CVD* cardiovascular disease(s), *GnRH* gonadotropin-releasing hormone, *PSA* prostate specific antigen, *TNM* where T = tumor, N = nodes and M = metastasis.

^a^Yes: receiving cardiac therapy, diagnosis of ischemic heart diseases, stroke, or congestive heart failure 1 year before androgen deprivation therapy initiation.

**Table 2:** Cardiovascular outcomes (after 90 days of ADT initiation) among patients on GnRH antagonists and agonists by pre-existing cardiovascular disease.

	All			Pre-existing CVD^a^			No pre-existing CVD		
	GnRH antagonist *N* = 499	GnRH agonist *N* = 15,127		GnRH antagonist *N* = 167	GnRH agonist *N* = 3348		GnRH antagonist *N* = 332	GnRH agonist *N* = 11,779	
	*n* (%)	*n* (%)	*P* value	*n* (%)	*n* (%)	*P* value	*n* (%)	*n* (%)	*P* value
Receiving hyperlipidemia treatment	125 (25.1)	5183 (34.3)	<0.0001	61 (36.5)	1589 (47.5)	0.0057	64 (19.3)	3594 (30.5)	<0.0001
Receiving cardiac therapy	123 (24.6)	4960 (32.8)	<0.0001	56 (33.5)	1381 (41.3)	0.0478	67 (20.2)	3579 (30.4)	<0.0001
IHD, stroke or CHF	18 (3.6)	1758 (11.6)	<0.0001	3 (1.8)	410 (12.3)	<0.0001	15 (4.5)	1348 (11.4)	<0.0001
Vital status			0.3939			0.6542			0.6231
Alive	377 (75.6)	11,229 (74.2)		130 (77.8)	2570 (76.8)		247 (74.3)	8659 (73.5)	
CV-related death	7 (1.4)	348 (2.3)		2 (1.2)	76 (2.3)		5 (1.5)	272 (2.3)	
Other cause death	115 (23.0)	3550 (23.5)		35 (21.0)	702 (21.0)		80 (24.1)	2848 (24.2)	

*ADT* androgen deprivation therapy*, CHF* congestive heart failure, *CV* cardiovascular, *GnRH* gonadotropin-releasing hormone, *IHD* ischemic heart disease.

^a^Pre-existing CVD: receiving cardiac therapy, diagnosis of ischemic heart diseases, stroke, or congestive heart failure 1 year before androgen deprivation therapy initiation.

In the sentence beginning with “Risk groups defined by National Comprehensive Cancer Network...” value “20 ng/ml” has been changed to “>20 ng/mL”.

In this article and insertion to the caption to Fig 1 has been made. In the sentence beginning with “Survival curves...” it now reads as “Survival curves showing MACE-free survival probability (significance of difference tested by log rank test)...”.

The original article has been corrected.

