# Correction to: Cardiovascular risk of gonadotropin-releasing hormone antagonist versus agonist in men with prostate cancer: an observational study in Taiwan

**DOI:** 10.1038/s41391-022-00596-5

**Published:** 2022-10-10

**Authors:** Yu-Hsuan Joni Shao, Jian-Hua Hong, Chun-Kai Chen, Chao-Yuan Huang

**Affiliations:** 1 Graduate Institute of Biomedical Informatics, College of Medical Science and Technology, Taipei Medical University, Taipei, Taiwan; 2 Clinical Big Data Research Center, Taipei Medical University Hospital, Taipei, Taiwan; 3 Department of Urology, National Taiwan University Hospital, College of Medicine, National Taiwan University, Taipei, Taiwan; 4 Institute of Biomedical Engineering, National Taiwan University, Taipei, Taiwan; 5 Division of Cardiology, Department of Internal Medicine, National Taiwan University Hospital, Hsinchu Branch, Hsinchu, Taiwan

**Keywords:** Cancer therapy, Prostate cancer

Correction to: *Prostate Cancer and Prostatic Diseases* 10.1038/s41391-022-00555-0, published online 03 June 2022.

In Table 4 of this article, the data in the risk of MACE and composite CV events headed receiving more than 6 months of ADT were mistakenly listed under the headed preexisting CVD, receiving more than 6 months of ADT and vice versa.

**Table 4:** Subgroup analysis estimating the risk of MACE associated with GnRH antagonist comparing with GnRH agonist.

GnRH antagonist vs GnRH agonist	MACE				Composite CV events			
	No of event	aHR^a^	(95% CI)	*P* value	No of event	aHR	(95% CI)	*P* value
Preexisting CVD, initial staging N=1 or M=1								
GnRH antagonist (*n* = 106)	34	0.98^a^	(0.66–1.45)	0.9071	3	0.16^b^	(0.04–0.38)	0.013
GnRH agonist (*n* = 1489)	621				188			
Receiving more than 6 months of ADT (GnRH antagonist ≥6 months vs GnRH agonist ≥6 months)								
GnRH antagonist (*n* = 286)	82	0.95	(0.74–1.22)	0.7023	15	0.30	(0.16–0.54)	<0.0001
GnRH agonist (*n* = 10615)	3780				1637			
Preexisting CVD, receiving more than 6 months of ADT (GnRH antagonist ≥6 months vs GnRH agonist ≥6 months)								
GnRH antagonist (*n* = 96)	24	0.64^**c**^	(0.39–1.05)	0.0757	3	0.12^d^	(0.03–0.49)	0.0032
GnRH agonist (*n* = 2006)	687				375			

*aHR* adjusted hazard ratio, *CV* cardiovascular, *GnRH* gonadotropin-releasing hormone*, MACE* major adverse cardiovascular event (ischemic heart disease, stroke, congestive heart failure or CV-related death).

preexising CV risk: receiving cardiac therapy, diagnosis of ischemic heart diseases, stroke, or congestive heart failure 1 year before androgen deprivation therapy initiation.

^a^aHRs were estimated using cox model adjusted for age, receiving chemotherapy, radiation therapy, antiandrogen, abiraterone, and enzalutamide.

^b^aHRs were estimated using the Fine and Gray competing risk model adjusted for age receiving chemotherapy, radiation therapy, antiandrogen, abiraterone, and enzalutamide.

^c^aHRs were estimated using cox model adjusted for age, cancer stage, receiving chemotherapy, radiation therapy, antiandrogen, abiraterone, and enzalutamide.

^d^aHRs were estimated using the Fine and Gray competing risk model adjusted for age, cancer stage, receiving chemotherapy, radiation therapy, antiandrogen, abiraterone, and enzalutamide.

The original article has been corrected.

Page 4: Last sentence before the section of Survival analysis.

In patients with pre-existing CVD and receiving ADT for ≥6 months, a 70% lower risk of composite CV events was determined in GnRH antagonisttreated patients than GnRHa-treated patients (aHR 0.30; 95% CI, 0.16–0.54; *p* < 0.0001; Table 4).

