# Supplementary material for: Cardiovascular risk of gonadotropin-releasing hormone antagonist versus agonist in men with prostate cancer: an observational study in Taiwan
Source: Prostate Cancer Prostatic Dis. 2022 Jun 3;26(4):722–9. doi: 10.1038/s41391-022-00555-0 (PMC10638084; doi:10.1038/s41391-022-00555-0)
Supplement: Supplementary file 1 — Supplementary table 1 [file 41391_2022_555_MOESM1_ESM.docx]

**Supplementary Table 1** ICD and ATC codes used for study variables data extraction

| **#** | **Variables** | **ICD-9** | **ICD-10** | **ATC** | | **Item/description** |
| --- | --- | --- | --- | --- | --- | --- |
| **1** | **Death** |  | PCa: C61  CV: I00–I99 |  |  |  |
| **2** | **GnRH agonist** |  |  |  | L02AE02 | Leuprorelin/leuprolide |
|  |  |  |  |  | L02AE03 | Goserelin |
|  |  |  |  |  | L02AE04 | Triptorelin |
| **3** | **GnRH antagonist** |  |  | L02BX | L02BX02 | Degarelix |
| **4** | **Concomitant therapy** |  |  |  | G03HA01 | Antiandrogens |
|  |  |  |  |  | L02BB03 | Bicalutamide |
|  |  |  |  |  | L02BB04 | Enzalutamide |
|  |  |  |  |  | L02BX03 | Abiraterone |
| **5** | **Hypertension** | 401–405 | I10–I15 | C02, C03, C07, C08, C09 |  | Hypertension (≥2 diagnoses within 6 months) |
| **6** | **Cardiac therapy** |  |  | C01 |  | Cardiac therapy (at least≥ 2 prescriptions for drugs ATC code: C01) |
| **7** | **Acute myocardial infarction and other forms of ischemic heart disease** | 410–414 | I21–I25 |  |  |  |
| **8** | **Stroke** | 433, 434, 436 | G450  G458  G459  I630  I632  I633  I634  I635  I64  I650  I651  I652  I653  I658  I659  I668  I669 |  |  |  |
| **9** | **Congestive heart failure** | 428 | I50 |  |  |  |
| **10** | **Diabetes** | 250 | E11 |  |  | Diabetes (≥2 diagnoses within 6 months) |
| **11** | **Dyslipidemia** | 272 277.7 | E78 | C10 |  | dyslipidemia (≥2 diagnoses within 6 months) |

*ATC* anatomical therapeutic chemical classification system, *GnRH* gonadotropin-releasing hormone, *ICD* International classification of disease.
